# Dr. Reeder on Diseases of the Heart

**Published:** 1821-09-01

**Authors:** 


### XIII.

*A Practical Treatise on the Inflammatory, Organic, and Sympathetic Diseases of the Heart; also, on Malformations of the Heart, Aneurism of the Aorta, Pulsation in Epigastrio, &c. &c.* By Henry Reeder, M.D. Member of the Medical and Chirurgical Society of London; and Extraordinary Member of the Royal Medical Society of Edinburgh. London, 8vo, 1821; 276 pages.

For our present improved knowledge of the diseases to which the internal organs are liable, we are principally indebted to the practice of necrotomy, conducted by men distinguished for their zeal, and animated by a spirit of genuine philosophic research. To this may perhaps be added the modern fashion of writing exclusively on one particular viscus, which, in return for the multiplication of books and the fatigue of perusing them, has certainly contributed to improve our nosologic and therapeutic science. In the present dormant state of medical lexicography,* compilations become necessary; and Dr. Reeder has endeavoured to render the profession a service by collecting the scattered remarks on the different diseases, which form the subjects of his book.

I. *Carditis.* The heart, Dr. R. observes, is liable to inflammation of the acute, sub-acute, and chronic characters, in which the pericardium is commonly more or less involved, and often the lungs. The resemblance of carditis to inflammation of the lungs probably induced Linnéus to omit the former in his arrangement. Dr. R's description of the acute form of carditis is similar to that of most nosologists.

The sub-acute form differs chiefly from the acute by the symptoms being more moderate. When it ends in death, the *facies hippocratica* is observable.†

The chronic species of carditis being the most difficult to detect, and having of late excited particular notice, we shall not do justice to the author nor to our readers, without representing his description in his own words.

"Some degree of febrile affection, a small, quick, and irregular pulse; and a peculiar jarring sensation communicated to the hand when placed over the situation of the heart. Palpitation of this organ is generally absent, but when it does occur, it is inconsiderable in point of extent. There is usually no pain, nor even sometimes any uneasiness, in the region of the heart; while, at other times, the latter, or a slight degree of obtuse fugitive pain, may be present. More commonly, however, a pain, more or less acute and perhaps fixed, is experienced in some part of the abdomen, most frequently in the epigastric or hypogastric region; and when it is seated in the latter situation, it is oftentimes attended with a suppression of urine. Sometimes there is a sense of beating in the head;

---

* We are astonished that a second edition of Dr. Parr's Medical Dictionary, or one on a more comprehensive plan, has not made its appearance in this country.—Rev.

† By some accident the word *Hippocratic* is printed with a small *h*. We trust we need not inform our author that all adjectives, derived from proper names, should be written with a capital letter at the beginning of the word. This mark of respect is peculiarly due to the father of medicine.—Rev.

and, in the more advanced stage of the disease, a pulsation in the epigastric region frequently takes place, and having, in some degree, the appearance as if it arose from a throbbing tumour, and evincing, as will afterwards be shewn, that the pericardium is then adherent to the heart. Obstinate vomiting is occasionally acceded. At length dropsical affections supervene, as œdema of the face and extremities, and effusion into the cavity of the thorax; the breathing then becomes more difficult and laborious; the countenance assumes an anxious and bloated aspect; delirium not unfrequently comes on; and the patient, after much suffering, of longer or shorter duration, sinks into dissolution." 25.

Cooks and laundresses are, we think, very subject to this complaint, which is generally accompanied with a deadly paleness, yet bloated appearance in the face, and a serous effusion in the cellular substance of the eye-lids.

Carditis is often conjoined with inflammatory affections of some of the other contents of the thorax.

The various morbid appearances, which the heart presents on dissection, have been described by Baillie, Morgagni, Corvisart, Bonetus, and others, and are briefly noticed by the author.

*Rheumatic Inflammation of the Heart* is the result of metastasis, and is either complete or partial. In the former case the inflammation and pain in the extremities are entirely removed; in the latter they are but little, or not in any degree diminished. When the cardiac affection is mild, a palpitation only without pain is perceptible. The consequence of a long-continued rheumatic affection of the heart is generally an enlargement of that organ: whence violent palpitation, short cough, dispnœa, quick and irregular pulse, hydrothorax, and anasarca, or ascites. The post mortem appearances produced by metastatic carditis are an adhesion of the pericardium to the heart, which, as we have just observed, is generally much enlarged; inflammation or congestion of the membrane lining the cavities; or an inflammatory redness in the cuticular coats of the arteries and veins to a considerable distance. Dr. Reeder has seen this disease succeeded by chorea, which speedily destroyed his patient. In one case of rheumatic carditis, which came under our notice, besides the usual symptoms, there were present profuse perspiration soon after the commencement, with universal tremor of the muscles. By copious bleeding, hydrargyri submur. and opium, our patient, an old man, was restored. He had some time afterwards a relapse from obstructed perspiration, and died. We found the pericardium adhering to the heart, by means of coagulable lymph, so closely, that all distinction between the two parts was lost. The heart was white and enlarged, and the pleuræ adherent in several places. We are inclined to believe, that in weak constitutions, in consequence of the metastasis of rheumatism and perhaps of gout, the heart is subject to severe congestion, producing symptoms similar to those of carditis, excepting that acute pain is absent. We met with two cases in one family: one of them, a delicate female, who had suffered many tiresome attacks of acute rheumatism, died with cardiac congestion on the fourth day, without experiencing any urgent pain or manifest reaction; the other, a corpulent but feeble man, was restored by copious bleeding and calomel and opium, acute rheumatism having made its appearance. In the former case bleeding and other active measures were not permitted to be made use of, for fear of *debility*; and necrotomy was forbidden.*

*Inflammation of the arteries and veins*, when it extends to the heart, produces carditis, attended generally with delirium.

*Treatment of Carditis.* Dr. R. recommends the most vigorous antiphlogistic remedies and regimen, together with blisters, and in the sub-acute and chronic species the employment of mercurial preparations in addition to the other means. Our own experience justifies us in urging the prompt administration of hydrargyri submurias and opium, immediately after bleeding, in the most acute and active species of carditis, as well as in the other forms of the disease.

2. *Angina Pectoris.* Dr. Reeder's account of the causes and symptoms of this formidable and incurable disease is taken from the best authors in general. The sensations of the patient are in some measure modified by the situation and nature of the morbid change of structure. In one of our patients, an old and celebrated sportsman, extreme tenderness, of small extent, seated in the middle of the dorsal spine, uniformly attends every attack. We are at present uncertain from what source his disease originates. Sympathetic angina is, we believe, generally occasioned by some derangement in the functions of the digestive organs; and we have known several persons who have not had more than one or two paroxysms. One gentleman had angina

* It is remarkable that another female in the same family labours under chronic carditis. Rev.

from derangement in the stomach, when he was fifty years old, and during the last ten years has escaped.

The concretions formed about the heart and large arteries consist of phosphate of lime; and, as those produced by gout are composed of urate of soda, Dr. R. thinks that there can be no affinity between gout and angina pectoris.

*Treatment.* In the intervals between the paroxysms much may be effected to prevent their accession, by the patient observing proper rules with respect to exercise and diet; and by avoiding exciting causes. Exercise, particularly that of equitation, should not be had recourse to, when the stomach is full. Dr. R. advises his patients to drink water and to eat sparingly; to keep the bowels open; to regulate the temperature of the body by clothing, and to avoid heated rooms and an impure atmosphere. Occasional plethora should be removed by bleeding in the recumbent position, or by cupping, and its recurrence prevented as much as possible by the almost exclusive use of farinaceous food. Issues have been much praised, in the thighs or arms, and we have often made trial of them, as well as of the tartrite of antimony, to excite a pustular eruption; but we have found an occasional blister answer every purpose, with much less inconvenience to the patient.

While the paroxysm is present, Dr. R. advises the abstraction of a few ounces of blood, the patient having been laid in the horizontal position. He has an objection to the use of internal stimuli, unless the heart appear unable, after the lapse of some time to regain its usual action, when weak wine and water, a small quantity of æther or spirit of ammonia diluted, may be given. One of our patients experiences immediate relief by strong brandy and water. Should not these succeed, Dr. R. would apply a blister over the cardiac region, and immerse the arm, when much affected, in hot water, and afterwards direct it to be rubbed with some stimulant and anodyne liniment. Opium may be given with advantage in a protracted paroxysm, and the author observes, that this medicine or the extract of hyoscyamus is the best adapted to prevent noctural attacks, when given at bed-time. He repeats that diffusible stimuli are almost totally inadmissible; and should the syncope remain an undue length of time, it will be necessary to transmit electric or galvanic* shocks through the region of the heart, and

* We are sorry that we have occasion to repeat our remarks on the inaccuracy and disrespect in writing adjectives derived from proper names with small letters at the beginning: as we find in the word *Galvanic.*—Rev.

to inflate the lungs by proper bellows, so as to establish an artificial, pulmonary, and aortal circulation.

Twelve cases of angina pectoris, already published in different medical works, are detailed. These were all found to have originated in an ossification of the coronary arteries. Several other cases, proceeding from other causes, are also related; but it seems uncertain whether the coronary arteries were carefully examined in every instance.

3 *On Change in Structure of the Valves of the Heart and large Arteries, and on Morbid Contraction of the different Apertures to which they are attached; also, on Alteration in the Structure of the Muscular Substance of the Heart itself, &c.* Our limits will not allow us to follow the author in his circumstantial account of the various appearances presented by a view of the morbid state of the heart and large vessels. He does not agree with Bichat, who thought that the right auriculo-ventricular and pulmonary arterial apertures, and the right tricuspid and semilunar valves were never subject to organic derangement.

When the valves are enlarged, or the apertures contracted on the left side of the heart, a state of great distress is liable to be induced by any exciting cause.

"Palpitation of the heart is experienced, which is sometimes so considerable, as to be audible to the by-standers, or to shake the thorax, or even the greater part of the body. The pulse at the wrist is hard, quick, irregular, and intermittent, and the length of its intermission varies a good deal, being, in some instances, so long, that two, three, or four strokes ought to have taken place; it is oftentimes very weak, small, and contracted, while the pulsations of the heart are performed in a violent, irregular, tumultuous, or fluttering manner. In some cases, moreover, two or three strokes of the heart are felt to only one of the radial artery, hence the number of pulsations of the heart, in the course of a minute, exceed greatly those of the pulse at the wrist; and occasionally it is different in one wrist from what it is in the other. Sometimes the heart makes one full stroke against the ribs, and is then followed by two or three indistinct pulsations. On some occasions, when the ear is placed near the chest, a hissing or rustling sort of noise may be heard therein, as of the rushing of water, or as if a fluid were passing through too narrow an orifice; the patient himself is sometimes able to hear this peculiar noise." 150.

Dyspnœa is also present, and the countenance, and sometimes the whole surface, assumes a livid aspect; indicating a general fulness of the venous system. Oppression is felt in the chest, attended with anxiety, and often with pain under the left side of the sternum, extending towards the spine, or the shoulders, or arms, and producing a sense of numbness in the two last situations. A cough, expectoration, and hæmoptysis, sometimes attend, also a pulsation of the jugular veins, snycope, head-ache, vertigo, mental irritation, or delirium. The liver becomes sometimes enlarged, and as the disease advances, dropsical effusions follow. The patient at length is destroyed by syncope or by extreme exhaustion.

Enlargement of the valves, or contraction of the apertures in the right side of the heart, produces the same phænomena as we have just stated to follow similar organic lesions in the left; excepting that the dyspnœa is slight, and hæmoptysis is absent.

When the valves permit the reflux of blood, symptoms, similar to those resulting from the organic affections already mentioned, present themselves.

The obstruction which the blood experiences in its return from the head, is apt to occasion apoplexy, paralysis, or epilepsy; and a similar retardation in the reflux of the blood is followed by hæmorrhage from the nose, lungs, intestines, &c. Enlargement of the liver is also produced by the same cause.

*Rupture of the Heart* has been known to happen where it had not been previously diseased. This accident, however, is generally owing to a morbid tenuity of the walls of the heart, which are unable to resist the pressure against them, occasioned by any great difficulty in the transmission of the blood. The left cavities are more liable to it than the right, and the patient is either instantly or soon destroyed.

*Change of Structure in the Substance of the Heart.* The ventricles have been found converted into an osseous or cartilaginous substance, and so indurated as to be incapable of performing their functions. The auricles, being less liable to be affected, acquire an increase of substance and of muscularity, and thus, with the aid of the arteries, carry on the circulation. When the right ventricles are thus diseased, a pulsation in the jugular veins and in the epigastrium is established; and when the left is in the same state, dyspnœa and hæmoptysis present themselves.

*Treatment.* The several diseases we have just mentioned require the same rules to be observed by the patient, as have been laid down for the treatment of angina pectoris. Very little can be expected from medicine.

Dr. Reeder relates two cases, exhibiting the morbid states to which we have been alluding; also two, which have been published by Mr. Abernethy, and one by Mr. John Hunter.

4. *Enlargement of the Heart, &c.* When this disease is simple and uncomplicated, arising solely from the addition of muscular substance, we have repeatedly seen the best effects result from occasional bleeding and digitalis, as recommended by the author. We have always prescribed the tincture, and gradually augmented the dose, until an effect has been produced on the circulation.

Some of the other varieties of cardiac enlargement are described, and their nature and symptoms explained, with tolerable accuracy. Rules are also laid down to assist our diagnosis. Two cases are appended by the author, and two others transcribed from the writings of Burns and Haller. Our author, however, has shewn no very good judgment in the compilation of this part of the work, by passing over several recent writers, particularly continental, on organic diseases of the heart.

We have bestowed on this article a much greater space than we had intended at its commencement; and must therefore conclude with a bare enumeration of the remaining subjects, which form a considerable portion of the book. These subjects are *diminution in the size of the heart, adhesion of the pericardium, polypi in the cavities of the heart, sympathetic affections and malconformations of the heart, hydrops pericardii, aneurism of the thoracic portion of the aorta and pulsation in epigastrio.*

The precise nature of pulsating tumours in the epigastrium it is exceedingly difficult to ascertain. This is candidly acknowledged by Morgagni,* who remarks that it is not equally easy to avoid being deceived sometimes, when a body of some extent, which strikes against the hand, may either be a dilated artery, or a tumour lying upon an artery which is not dilated. A suppurated jugular gland was supposed by himself to have been an aneurism; and Tabarranus† mentions how greatly he was deceived by a pulsatory tumour in the epigastric region, of the size of a fist, which he imagined to be a true aneurism; but which proved, on opening the body, to be a scirrhous tumour in the mesentery. Severinus had also a patient, in whom there was a pulsation in the neck, which puzzled many sur-

---

* Morgagni de Causis et Sedibus Morborum. Epis. 39, Art. 20.

† Obs. Anatom. Ed. 2, n, 9.

geons, and induced them to say it proceeded from aneurism. It was however discovered, as Severinus had foretold, to be occasioned by the action of the carotid artery under a bronchocele. An enlarged and tuberculated liver will produce the external appearance and violent throbbing of a large aneurism in the epigastrium; and a remarkable case of this kind, accompanied with some interesting observations, has been published by Mr. J. M. Coley, in the Medical and Physical Journal, Vol. xviii. p. 485.

In conclusion, we are sorry to be obliged to remark, that we are not very well satisfied with the present Treatise. We do not observe many marks of personal observation or experience, and in the compilation from others, Dr. Reeder has often neglected to avail himself of the *best* information, while he swells the work with the most *common-place* matter from books in the hands of every one. We need only instance his neglect of the invaluable researches of Laennec on Organic Diseases of the Heart, besides many other very valuable observations and cases published on the Continent. Should the work come to a second edition, Dr. Reeder will do well to attend to these; and as it is evidently a compilation, there is no excuse for not selecting the best materials from the most authentic sources.

